# Development and Evaluation of Bluetooth Low-Energy Device for Electronic Encounter Metrics

**DOI:** 10.6028/jres.126.043

**Published:** 2022-01-20

**Authors:** Kathryn E. Keenan, José Aumentado, Harold Booth, Kimberly A. Briggman, Mikail Kraft, Michele N. Martin, René C. Peralta, Angela Y. Robinson, Krister Shalm, Michelle S. Stephens, Emily A. Townsend, Sae Woo Nam

**Affiliations:** 1 National Institute of Standards and Technology, Boulder, CO 80305 USA; 2 National Institute of Standards and Technology, Gaithersburg, MD 20899 USA; 3 3Department of Physics, 390 UCB, University of Colorado, Boulder, CO 80309 USA; 4 Joint Quantum Institute, University of Maryland, College Park, MD 20742 USA

**Keywords:** Bluetooth low energy, BLE, COVID, encounter metrics, encounter notification

## Abstract

The coronavirus disease 2019 (COVID-19) pandemic led to the need for tracking of physical contacts and potential exposure to disease. Traditional contact tracing can be augmented by electronic tools called “electronic contact tracing” or “exposure notification.”. Some methods were built to work with smartphones; however, smartphones are not prevalent in some high-contact areas (e.g., schools and nursing homes). We present the design and initial testing of low-cost, highly privacy preserving wearable exposure notification devices. Several devices were constructed based on existing hardware and operated independently of a smartphone. The method (devices and analyses) was not able to reliably use the received signal strength indicator (RSSI) as a proxy for distance between pairs of devices; the accuracy of RSSI as a proxy for distance decreased dramatically outside of the idealized conditions. However, even an imperfect device could be useful for research on how people use and move through spaces. With some improvement, these devices could be used to understand disease spread and human or animal interaction in indoor environments

## 1 Introduction

The coronavirus disease 2019 (COVID-19) pandemic has prompted the development of electronic tools that can be used to identify close contacts and potential exposure to disease [1]. These tools, typically referred to as “electronic contact tracing” or “exposure notification,” use short-range electronic ranging methods such as short-range radio communication techniques (*e.g.*, Bluetooth^1^1 Certain commercial equipment, instruments, or materials are identified in this paper to foster understanding. Such identification does not imply recommendation or endorsement by the National Institute of Standards and Technology, nor does it imply that the materials or equipment identified are necessarily the best available for the purpose. low energy [BLE] [2]) or ultrasonic techniques [3] to determine the distance between two or more individuals. When the individuals are determined to be “too-close-for-too-long,” the encounter is recorded in a way that is designed to preserve anonymity. If one individual becomes ill, the individual or health officials are offered a privacy-preserving means by which to report the illness and allow electronic notification of the other individuals involved in a too-close-for-too-long encounter. Notified individuals may then choose to modify their behavior in some way, possibly by getting a COVID test, contacting a healthcare expert, or quarantining for some period of time [4]. These electronic tools can augment manual contact tracing by identifying unknown contacts of possible exposure and notifying exposed individuals. If used, these features could help to limit the spread of a contagious disease like COVID-19.

Several protocols for exposure notification were developed in 2020, including the Google Apple Exposure Notification (GAEN) system [5–6]. GAEN uses BLE signals recorded between two smartphones to estimate the distance between two individuals. The interface to the cloud is through the smartphone; exposure notification data are stored and processed on the smartphone. GAEN has been adopted in 40 countries worldwide and 26 states and territories in the United States [7]. However, it relies on smartphone technology that may not be accessible to all communities and raises privacy concerns among some users. In this work, we investigated the development of a low-cost Bluetooth® device to be used in various settings, especially where people do not have smartphones, *e.g.*, schools, nursing homes. Another key design goal was for the device and protocol to be highly privacy preserving, including being decoupled from the global positioning system (GPS) or location information, and for this reason, it was required to be separate from a user’s smartphone.

In this article, we present the design and initial testing of low-cost, highly privacy preserving wearable exposure notification devices.

## 2 Methods

We designed and constructed a device to be worn for encounter notification using Bluetooth signals to determine the proximity of other nearby devices. The Bluetooth low-energy systems measure a received signal strength indicator (RSSI), which we used as a proxy for distance. Each time two devices sensed that they were nearby, they generated a unique, privacy-preserving encounter identifier that was recorded by each device. The devices could then communicate with other hardware (phone, tablet, or computer) to receive commands to reconfigure the devices or upload recorded data. Here, we describe the hardware design, the device architecture, and the studies using the devices.

### 2.1 Hardware Design

One of the most important considerations in developing a low-cost exposure notification device is the implementation of the hardware to perform the tasks required. The following criteria were considered when selecting the Bluetooth chipset used: Bluetooth feature set, power consumption, interface to external peripherals, development environments available, and cost. In addition, to expedite development, we only considered Bluetooth chipsets that were already integrated into inexpensive development boards. Examples of such boards include but were not limited to the BBC Microbit, Adafruit nRF52840 feather, and Particle Gen3 Argon.

One of the challenges of using the Bluetooth RSSI as an estimator for distance is that variations in RSSI can also be a result of multipath interference (from multiple reflections between the transmitter and receiver) as well as interference from other wireless devices that share the same frequency band (*e.g.*, Wi-Fi). As a result, it would be highly desirable to access other Bluetooth features and information that could be used to help determine distance. One such feature is the Bluetooth channel used to measure the RSSI in decibels with reference to one milliwatt (dBm). A Bluetooth device can send advertising information using three Bluetooth channels (37, 38, and 39) and receive information in over 40 channels. When a Bluetooth device listens for advertisements, the standard peripheral interface does not contain the channel information. However, knowledge of the channel number at the listener may help with proximity estimation. For example, if it is recognized that some of the channels are experiencing destructive interference, resulting in an unusually low received power, an alternate channel could be used or prioritized in signal processing. Another feature available in BLE 5.1 and 5.2 is angle of arrival (AoA) and angle of detection (AoD). With Bluetooth radios that are able to implement AoA and AoD protocols, it may be possible to implement high-resolution ranging by measuring the relative phase shift of the radio signals as a function of Bluetooth transmission frequency [8–9].

Another way to overcome some of the shortcomings of proximity detection using Bluetooth is to add additional sensors to the environment. Examples of such sensors that might be useful are device orientation, light sensors, thermometers, humidity, and microphones. The combination of such sensors could be used to determine, for example, that two devices that appear to be close together by radio measurements are in fact separated by a wall. While the use of other sensors would enhance proximity information, it could come at a cost to privacy unless careful thought is put into the information that is ultimately recorded from the sensors.

**Fig. 1:**
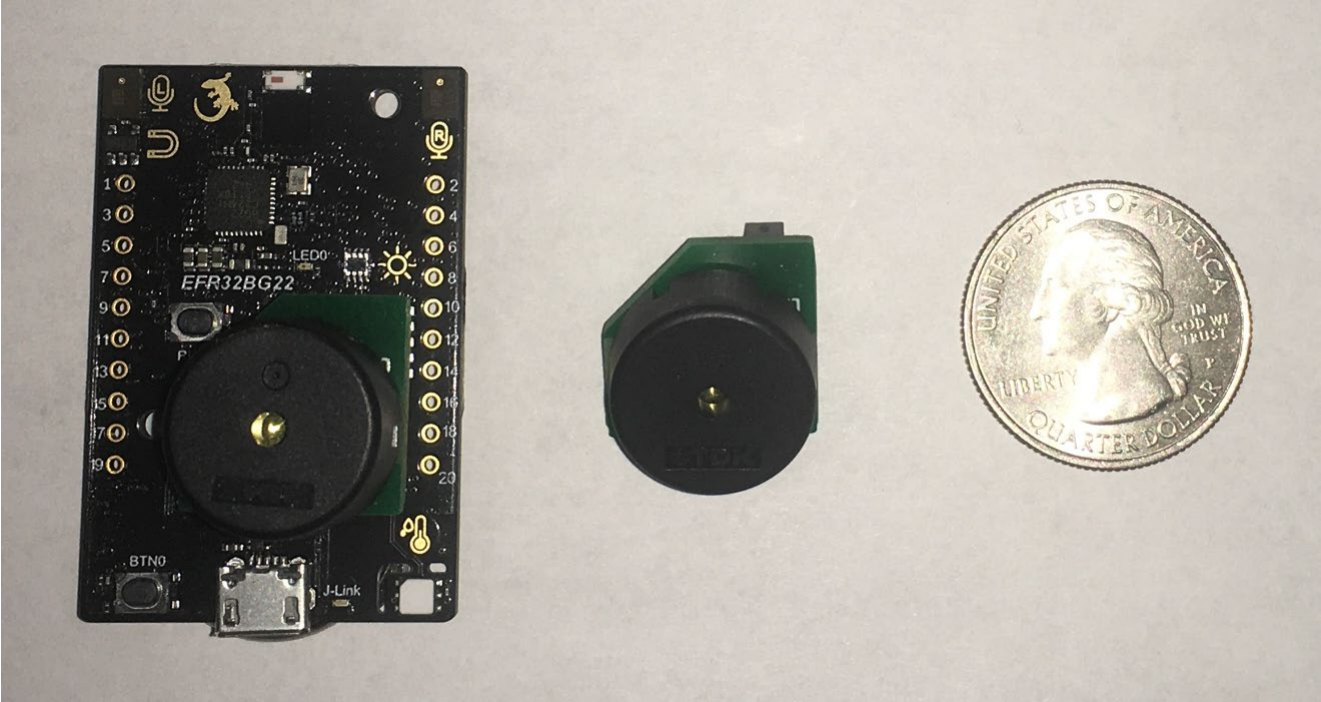
Photograph of Silicon Labs Thunderboard with a piezo buzzer mounted to an expansion socket on the left side. The piezo buzzer accessory board is pictured in the middle. A U.S. quarter is included in the figure for scale.

As a result of the requirements for more capable Bluetooth devices and access to other sensors, we used the Silicon Labs Thunderboard (SLTB010A EFR32BG22 Thunderboard Kit). Shown in the photograph in Fig. 1, this development board has a Bluetooth 5.2 radio capable of AoA or AoD as well as humidity sensor, thermometer, light sensor, magnetometer, stereo microphones, and a six-axis inertial motion sensor. It also has a coin cell battery holder and additional flash memory. .

#### 2.1.1 Architecture

Bluetooth technology has been developed for point-to-point interdevice communication over a range of less than 10 m. Bluetooth advertising and scanning functions are trivially adaptable for sensing proximity. Commercially available products such as Tile, iBeacons, and Eddystone Beacons have used Bluetooth for the past decade to determine whether two devices are nearby. In the Bluetooth Standard [10], a device can advertise its presence by transmitting a short message in one of three channels designated for advertising (channels 37, 38, and 39). A device can also scan for the presence of Bluetooth devices by listening for advertisements in these three channels. For battery-powered devices, time spent listening can severely impact battery life of the device. Consequently, in the GAEN protocol, the listening is usually set to occur once every 5 min, so that the phone battery life is not negatively impacted. As a result, it is possible for two devices using the GAEN protocol to “miss” each other’s presence because the advertising and listening may be out of sync. In the devices that we developed, the advertising rate and the scanning windows can be adjusted. To ensure that battery life would last at least one week, the default settings were programmed to

be advertisements broadcasted once every 200 ms and scans conducted once every 2.4 s for 200 ms. We did not vary the advertising and scanning strategies for effective real-world performance. However, this is something that could be the subject of further research.

**Fig. 2:**
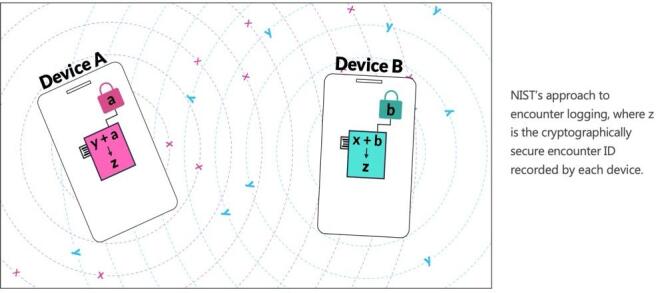
Visualization of the privacy-preserving encounter logging protocol.

A potential problem with Bluetooth advertisements used for proximity detection is the potential for lack of privacy. As a result, we implemented several features to protect user privacy [11]. The first is that we used a randomly generated Bluetooth media access control (MAC) address that was changed regularly and could potentially be changed as often as every encounter. Instead of using a changing identifier (*e.g.*, GAEN) for each device that was recorded when two devices were nearby, devices generated an “encounter ID” that was not able to be linked to the devices themselves. This shared secret key was generated by using the Diffie-Hellman key exchange using Curve 25519 [12–13]. To perform this key exchange, each device first generated a secret private key that was regenerated every minute. Second, using this private key, a public key was generated using the Curve 25519. Each device then broadcast its 32 byte public key using the Bluetooth advertisements. Finally, by combining the public keys heard over Bluetooth from nearby devices with its own secret private key, each device could generate a shared secret unique key to each encounter, the encounter ID (Fig. 2). This type of cryptographic key exchange and shared secret generation is well studied and tested, and it is used for securing web transactions today [14–16]. The private and public keys in a device are also changed regularly. In practice, the public and shared keys were computed using publicly available assembly language code to minimize computation time. The private keys were generated using a hardware random number generator built into the Bluetooth chip being used. The keys and MAC addresses could be changed every minute without detrimental effects on the performance. However, for this study, the keys and MAC addresses were only updated once a day; this was done to aid in the development of the techniques used to analyze the data.

Because of the limited power from a coin cell battery, these Bluetooth devices were not configured to connect to Wi-Fi or other types of networks. Instead, another device such as a phone, tablet, or computer was required to connect to this device via Bluetooth to configure it or retrieve data. For this study, we implemented Bluetooth services and characteristics so that a device could pair to the Bluetooth exposure notification device and configure the device or retrieve the recorded encounter metrics data.

##### 2.1.1.1 Device Testing

**Fig. 3:**
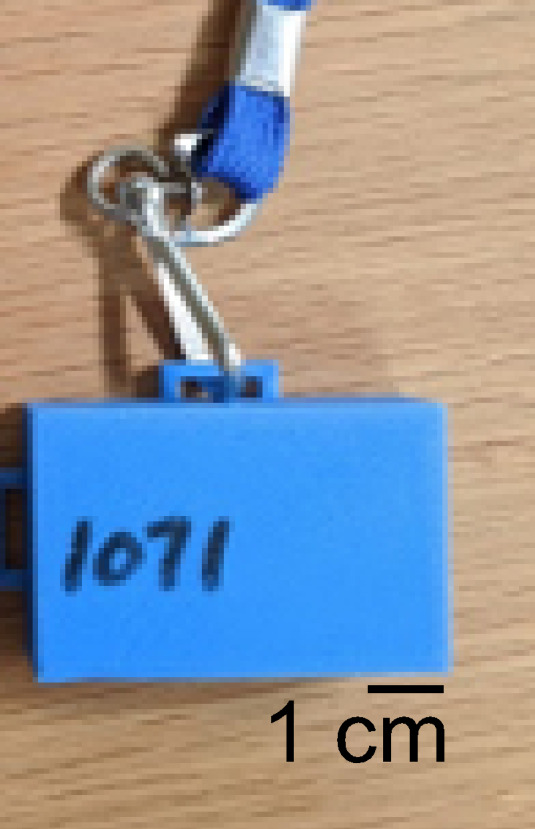
Photo of a device in a 3D-printed case and attached to a lanyard.

Seventeen participants in 10 households volunteered and consented to take part in the study of device performance in a household environment. This study was reviewed by the National Institute of Standards and Technology (NIST) Research Protection Office and determined to be “exempt human subjects research” as defined in 15 CFR 27, the Common Rule for the Protection of Human Subjects. Volunteers were given four Bluetooth devices per household to use for data collection. Each Bluetooth device was secured in a three-dimensional (3D) printer–created case that was attached to a lanyard (Fig. 3). A web interface was used for participants to log and annotate the collected data after uploading data from the device via a Bluetooth connection.

**Fig. 4:**
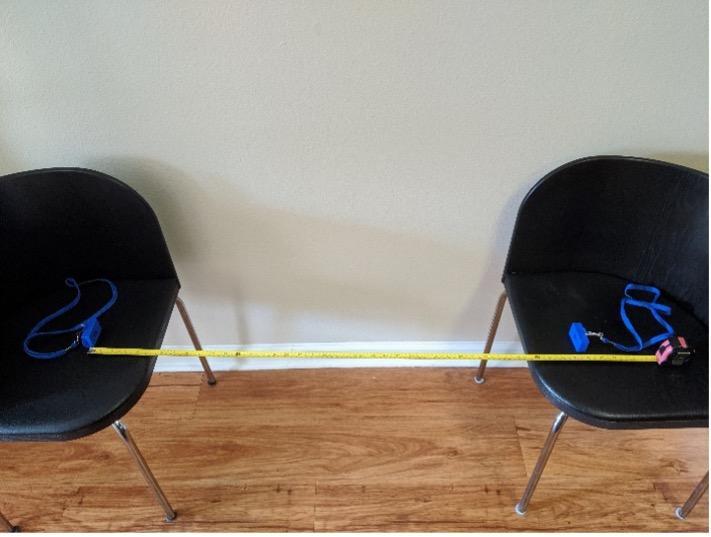
Photo of the baseline calibration testing configuration.

**Fig. 5:**
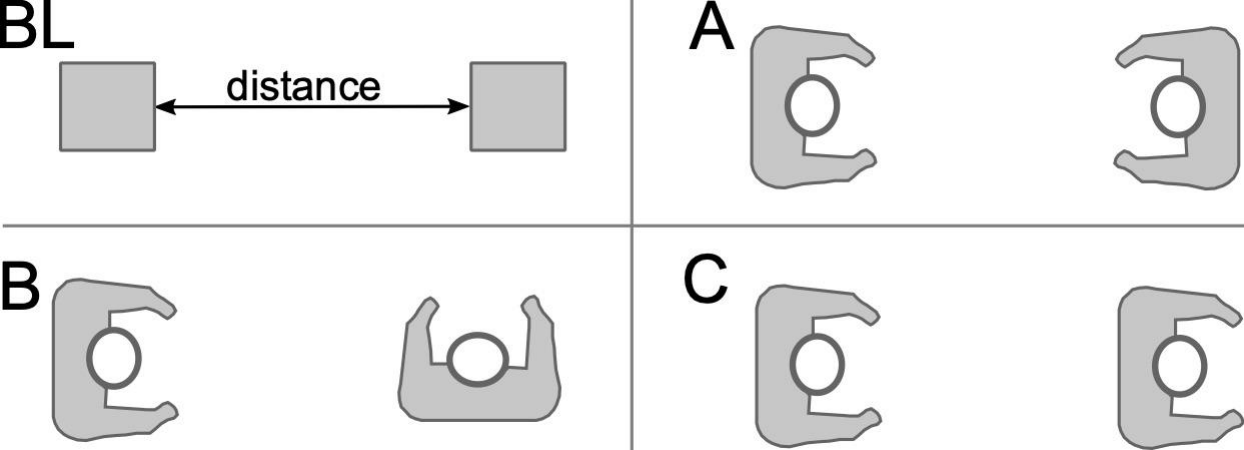
Diagrams of the calibration testing configurations. The device was worn on a lanyard around the neck with the device located on the chest. The device was not required to be held in a fixed orientation for the testing.

Participants were asked to use the devices in two different modes: calibration mode and encounter mode. For the first few (2–3) days of data collection, participants were asked to perform controlled tests with the devices in calibration mode. Calibration mode collected several radio signals per second, resulting in high-quality data that were collected over 5 min durations in a controlled manner. First, baseline (BL) tests were performed with devices placed on a flat surface at distances of 1 m, 1.5 m, and 2 m apart as shown in Fig. 4. Next, participants were asked to change the orientation of the devices and carry out the scenarios described in Table 1. All other devices that were not part of the test should have been placed in a household metal box, such as the oven or microwave oven, to minimize the chance for encounters with the devices being tested being recorded on these devices. Tests were performed with the devices at distances of 1 m, 1.5 m, and 2 m apart in each of the three configurations (A, B, and C) shown in Fig. 5. Tests could be carried out with two participants (each wearing one device) or with one participant (one device on the person and the other device placed so that it was at a comparable height). The participants recorded the calibration test type (BL, A, B, or C) and the distance between devices when logging the data in the web interface. Data were recorded for 5 min and used as a basis for algorithm development, to understand variability between devices, and to derive a receiver operating characteristic (ROC) curve.

**Table 1:** Scenarios used for calibration mode tests.

Configuration	Distance Between Devices	Optional Additional Distances Between Devices	Description
Baseline (BL)	1 m, 1.5 m, 2 m	Any other distances between 1 m and 4 m (*e.g.*, 2.5 m)	Devices facing each other on a flat surface (Fig. 4)
Configuration A			Volunteers wearing devices, or one device worn by a volunteer, and one positioned on a flat surface at same height (Fig. 5).
Configuration B			
Configuration C			

Next, participants were asked to use their devices in encounter mode to explore how the devices behaved in real, uncontrolled situations. Participants were asked to wear the devices via lanyards around their neck and collect data for at least 1 h every day as they carried out their normal routine. The devices recorded data only when explicitly set to record by participants. As a result, it was possible to receive a signal from a device but not have recordings from that device. When recording in encounter mode, radio signals were averaged over 1 min and were recorded once every 1 min. For households that consisted of more than one volunteer, each participant was asked to wear a device. All other devices were considered stationary and placed throughout the house in places where the participants would encounter them for greater than 15 min. When using encounter mode, participants were asked to log the times and the durations over which they thought they had an encounter with another device. Encounters were defined as being within 2 m of another device/participant for at least 15 min. The web interface (https://saewoonam.github.io/web_bt/) included a button that could be pressed when the participant was near another device to aid in logging. Participants also had the option of manually logging when encounters occurred using any method with which they were comfortable. The reported RSSI values are the mean of each of the channels 37, 38, and 39.

## 3 Analysis

Each device recorded RSSI values for multiple BLE channels as a function of time for each device pair in the set. RSSI, measured in dBm, is reported as negative numbers with numbers closer to zero corresponding to a stronger signal. Data categories recorded by the devices were: device time (time since switched on), the MAC address of the device recording data, RSSI, and the BLE channel.

For calibration, the data were filtered to find the device pair with the strongest mean RSSI, discarding data from other device pairs, as well as data points at the beginning and end of the files when the devices may have been turned on but the calibration configuration was not yet in place. Visual inspection of this data confirmed that each resulting data set likely represented a single configuration that was maintained for the entire duration of the test.

The filtered calibration data were used to estimate RSSI value for encounters less than 2 m. Analysis was performed using an ROC curve. First, the filtered calibration data were tagged as an actual encounter if the reported distance was less than 2 m (here, the reported distance was fixed for each calibration test). To test the utility of RSSI to accurately discriminate an encounter, we applied a sliding *M* (number of samples) of *N* (window size) window filter to the RSSI values, identifying a suspected encounter when at least *M* of the RSSI values in a window of *N* readings reached a particular threshold value [17]. We report ROC curves for this filter when *M* was 2 and *N* was 6 for all device sets in particular configurations and for all configurations combined. An ROC curve is a plot of the true positive rate (the likelihood that a suspected encounter has a reported distance less than 2 m) versus the false positive rate (the likelihood that it does not) for various threshold values. Each point on the plot represents a different threshold RSSI value between 0 dBm and −90 dBm. A random classifier will have a false positive rate equal to the true positive rate, and all points will lie on the “line of no discrimination” between (0,0) and (1,1), while better classifiers lie increasingly above and to the left of this line.

## 4 Results

### 4.1 User Calibration Tests

**Fig. 6:**
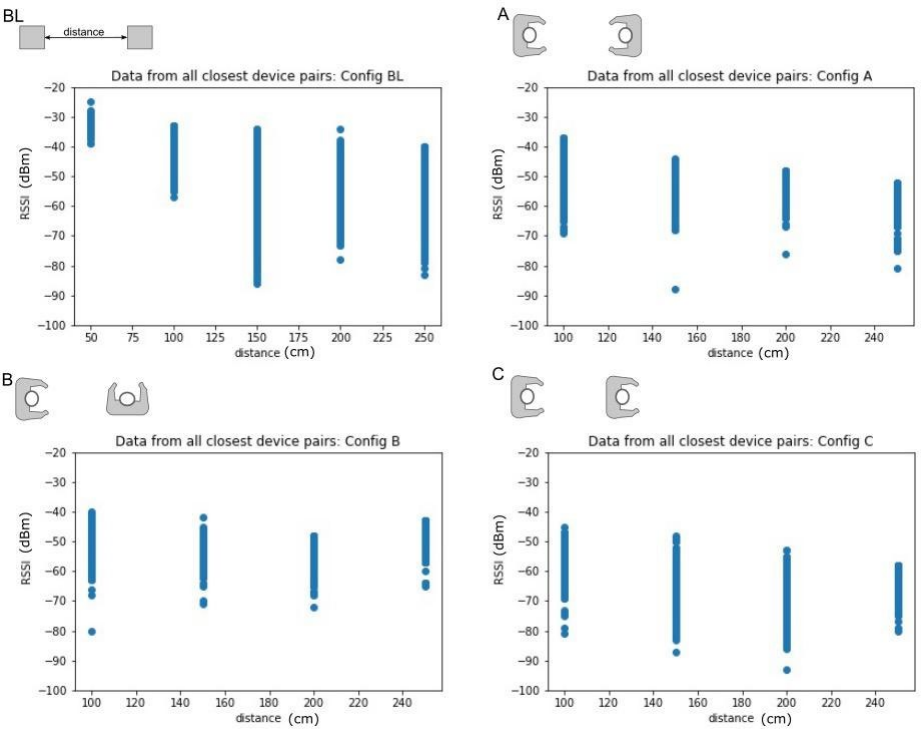
Example RSSI values at particular distances in the calibration configurations for channel 38 demonstrating some correlation between distance and RSSI, though it is dependent on the device orientation.

The user calibration data showed some correlation between the distance between the device pair in each configuration and the RSSI signal strength. Figure 6 shows the RSSI values from channel 38 for closest device pairs from all calibration tests across all participants as a function of the reported distance for the calibration. The spread of RSSI values for a given distance is a result of including a breadth of data.

**Fig. 7:**
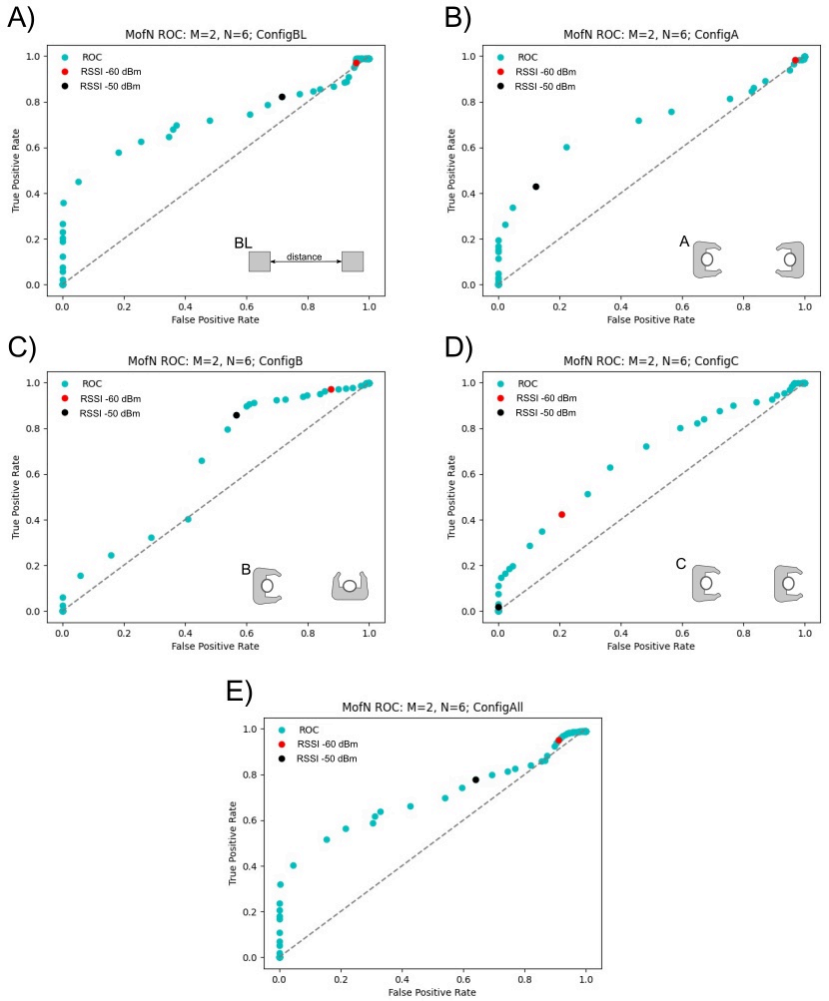
Calibration test results for channel 37 with *M* = 2 reads over a window of *N* = 6. The best result occurs with configuration A, where the users are facing each other.

**Fig. 8:**
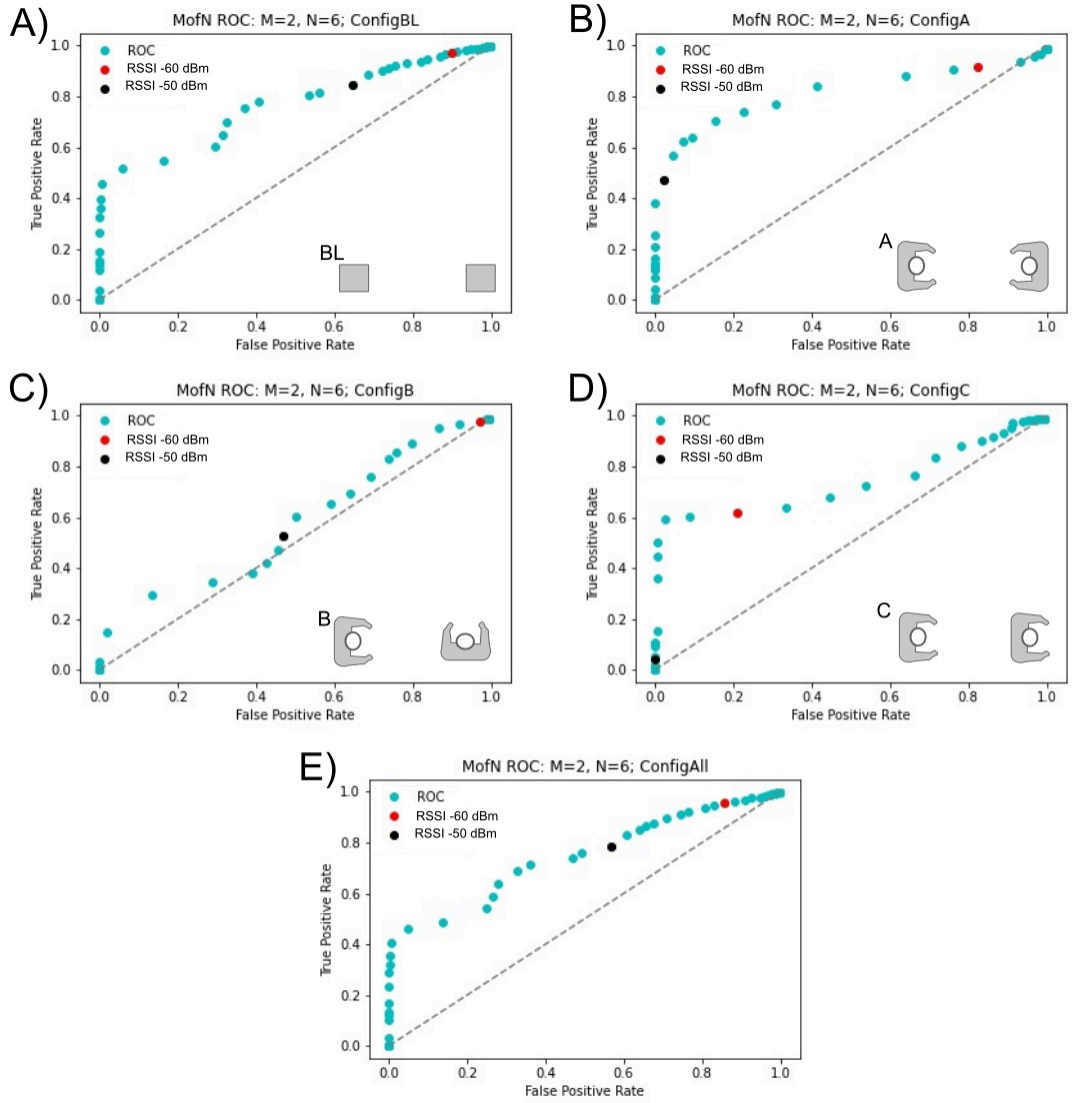
Calibration test results for channel 38 with *M* = 2 reads over a window of *N* = 6. The best result occurs with configuration A, where the users are facing each other.

**Fig. 9:**
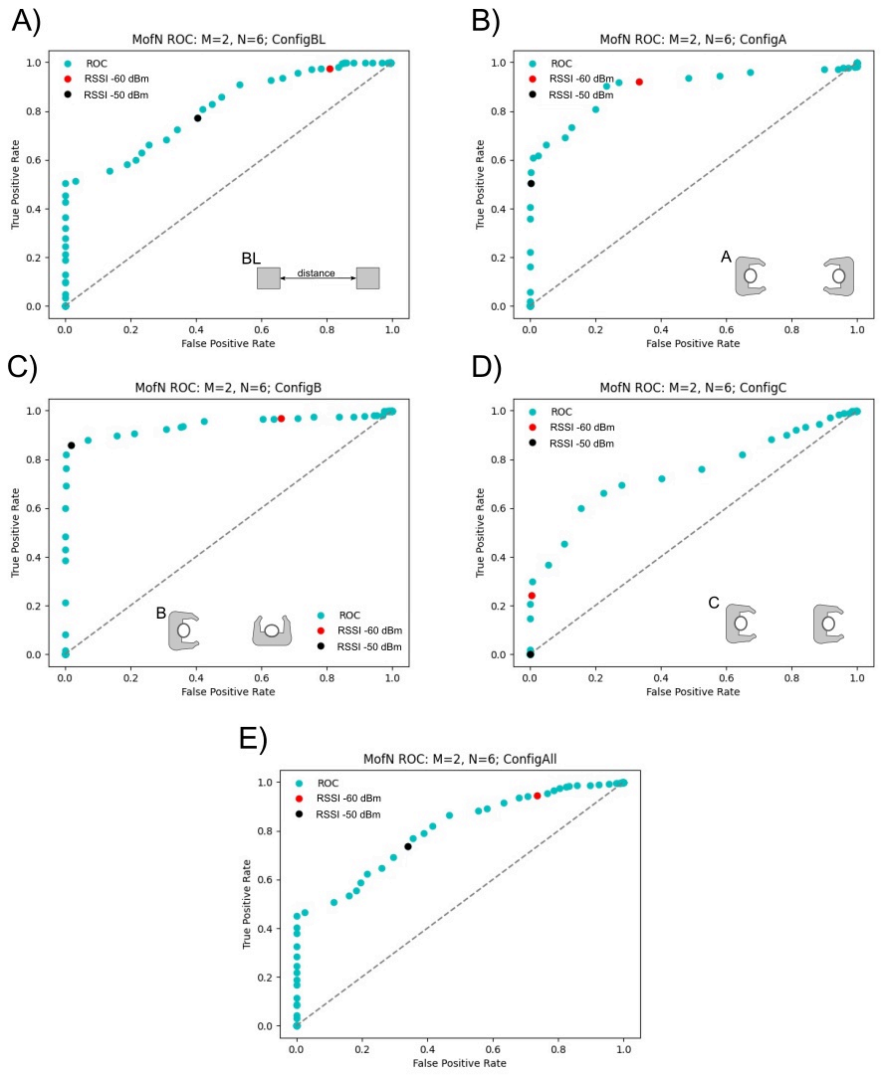
Calibration test results for channel 39 with *M* = 2 reads over a window of *N* = 6. The best result occurs with configuration A, where the users are facing each other.

Figures 7–9 show ROC curves using the *M* of *N* sliding window filter. All calibration test data from all volunteers were included to generate the ROC curves in these figures. All volunteers carried out measurements in the baseline configuration (BL) and then did tests in a subset of other configurations; this resulted in a larger sample size for the baseline configuration. As a result, the baseline configuration and the combination of all configurations are very similar. Additionally, there was a larger collection of data with the devices positioned at 2 m or less, rather than greater than 2 m. The analysis was performed for sliding window sizes *N* = 6 through *N* = 20 with *M* = 1 through *M* = 5. Given the high data sampling and the fixed positioning and of the calibration tests, there was minimal variability with different window sizes, *N*, samples, *M*. Data were recorded for the advertising channels 37, 38, and 39, and we report each channel individually. On these plots, the RSSI value of −50 dBm is a black dot, and the RSSI value of −60 dBm is a red dot; these are possible RSSI values to indicate an encounter at a distance less than 2 m.

When considering all calibration data, there are differences across channels 37, 38, and 39. Across these channels, channel 37 (Fig. 7) showed the worst prediction of distance from RSSI. For channel 38 (Fig. 8), the best results occurred in configuration A, when the devices were facing each other. Channel 39 was substantially different from the other two channels, in particular, for configuration B. For channel 39, configuration B, an RSSI value of −50 dBm was an excellent predictor of distances less than 2 m (Fig. 9C), whereas for channel 38, in configuration B (Fig. 8C), RSSI was unable to predict distances less than 2 m. Based on these results, channel 39 may be the most robust channel to use as a proxy for distance in these devices. The *M* of *N* plots in Figs. 7–9 suggest that, generally, an RSSI value of −50 dBm or −60 dBm is a reasonable proxy for an encounter of less than 2 m, because the ratio of the true positive rate to the false positive rate is far from 1 for these values.

#### 4.1.1 Encounter Mode Tests

In addition to the calibration studies, participants were encouraged to collect data in “encounter” mode, in which members of the household wore the devices on lanyards around their necks while moving around the home. Here, the RSSI is the mean value over channels 37, 38, and 39.

**Fig. 10:**
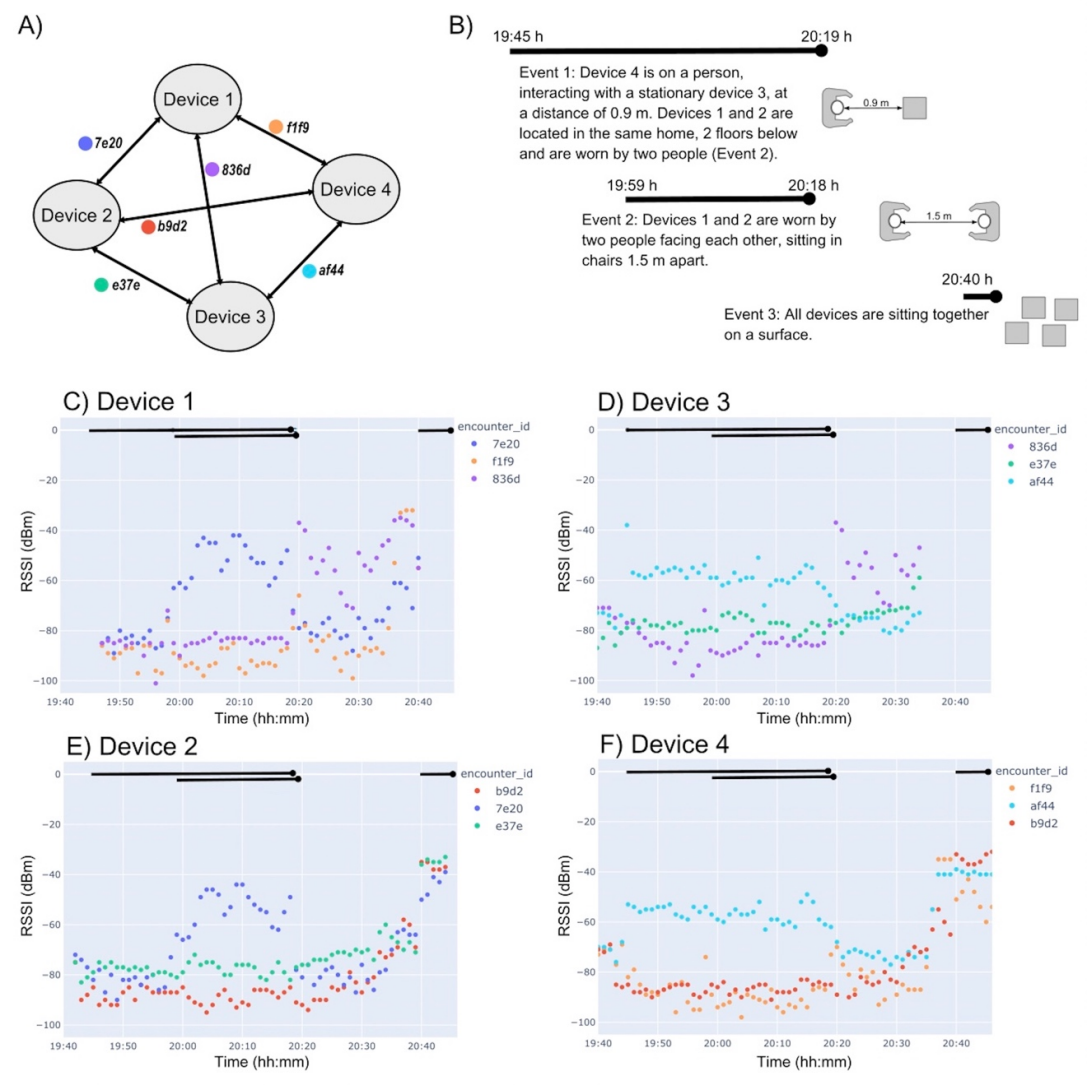
(A) The interactions between devices resulted in six encounter IDs. (B) The experimental events and conditions. (C) RSSI *vs*. time plot for device 1, which primarily interacted with device 2. (D) RSSI *vs*. time plot for device 3, which primarily interacted with device 4. (E) RSSI *vs*. time plot for device 2, which primarily interacted with device 1. (F) RSSI *vs*. time plot for device 4, which primarily interacted with device 3.

Figure 10 is an example data set submitted by a participant showing the interaction between four active devices located in the same home. Because the encounter ID was changed only once per day, we can see how these devices interacted over the course of an active 70 min window. The four devices interacted with each other, resulting in six encounter IDs: 7e20 (between devices 1 and 2), 836d (between devices 1 and 3), f1f9 (between devices 1 and 4), e37e (between devices 2 and 3), b9d2 (between devices 2 and 4), and af44 (between devices 3 and 4). The participant described the events and experimental conditions, and these are depicted in Fig. 10.

The first event from 19:45 to 20:19 h UTC is an interaction between device 4 on a person and stationary device 3 recorded as encounter ID af44. We note that the RSSI for device 4 is greater than that recorded by device 3, which could be due to the orientation of the device (and resulting antennae position) or due to the properties of the surface upon which the device was resting (reflection interference).

The second event from 19:59 to 20:18 h is a direct interaction between two people wearing devices 1 and 2 on lanyards around their necks and facing each other, 1.5 m (5 feet) apart. The resulting encounter ID, 7e20, RSSI is greater than −60 dBm on both devices, and the signal varies between −40 dBm and −60 dBm on both devices. The relative orientation of these devices is unknown. At the end of event 2, we notice that device 1 has an interaction with device 3, encounter ID 836d, that crosses the −60 dBm threshold for both devices. This event was not recorded by the participant, so we do not know the distance between devices.

The final event is only recorded in the data of devices 2 and 4. From 20:40 h until the end of data collection, all four devices were placed together on a surface, and we observe in Fig. 10, parts E and F, that the five recorded encounter IDs (b9d2, 7e20, e37e, f1f9, and af44) cross the −60 dBm threshold. There is no recorded signal between devices 1 and 3 (encounter ID 836d), since we only have the records for devices 2 and 4 for the time period after 20:40 h.

**Fig. 11:**
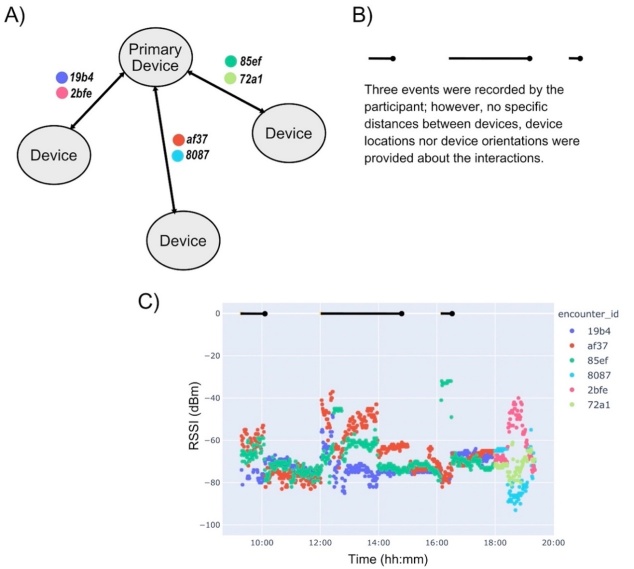
Example data from the encounter mode tests conducted within a home. (A) Details of the encounter IDs corresponding to the device pairs. (B) Event log. In this case, three encounters are indicated, and these are discussed in the text. (C) From this figure, we can observe the change in encounter ID name that occurred once per day at 18:00 h UTC. The single change in encounter ID per day was done for ease of analysis of the acquired data.

In a different set of household tests with four devices, we have the recorded signals from one of the four devices (the primary device), shown in Fig. 11. While this participant indicated when encounters occurred, there was no detailed log describing the encounters. The participant indicated three encounters less than 2 m, and the RSSI values did not always cross the −60 dBm threshold, a proxy for distances less than 2 m. The first encounter is indicated from approximately 9:00 h until approximately 10:00 h, and the encounter ID af37 RSSI is centered around −60 dBm. The participant did not record the dongles that were in proximity. Between 10:00 h and 12:00 h, the RSSI values were well below −60 dBm, accurately indicating no encounter (a true negative).

In Fig. 11, the second indicated encounter begins at 12:00 h and ends just before 15:00 h. In this window, there is no RSSI value continuously greater than −60 dBm; only encounter ID af37 exceeds that threshold for any substantial amount time (*e.g.*, 15 min). For encounter ID af37, the RSSI value drops below −60 dBm at 14:00 h, well before the indicated end of the encounter. Without details from the participant on distance between devices, it is not possible to tell which of these recorded RSSI values are true positives, true negatives, false positives, and false negatives.

The final encounter in Fig. 11 begins near 16:10 h and has an obvious RSSI signal above the −60 dBm threshold for encounter ID 85ef. In this case, we do not have an indication from the participant of the experimental setting or actions, so we cannot determine why the signal was obvious in some settings but not others, nor can we directly relate the measured RSSI to actual distance between the devices. Note, at 18:00 h, there are three new encounter IDs (8087, 2bfe, and 72a1). These are not new devices interacting; rather, this is the change in encounter ID name that occurred once per day.

**Fig. 12:**
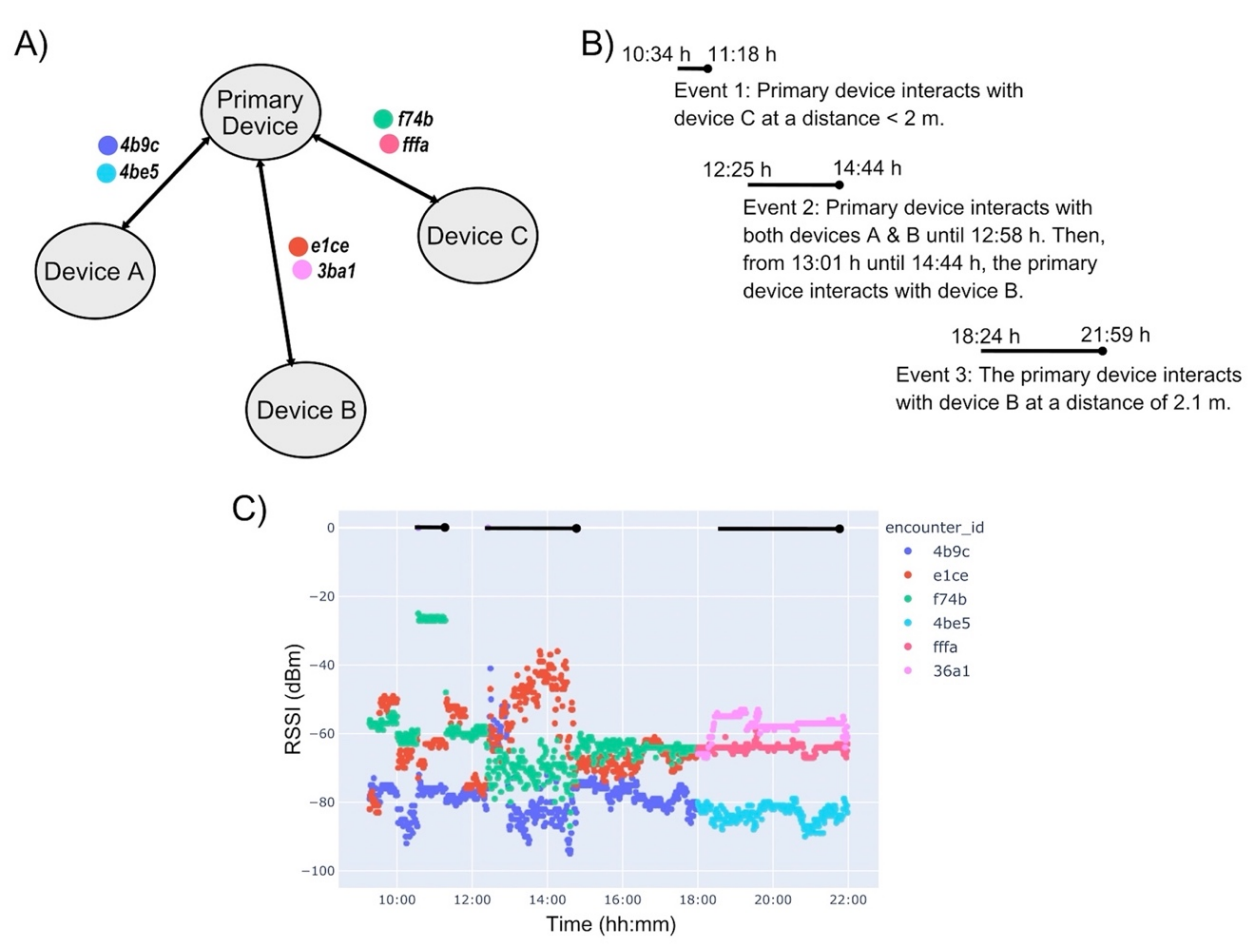
Another set of example data from the encounter mode tests conducted within a home. (A) Details of the encounter IDs corresponding to the device pairs. (B) Event log. (C) Here, two encounters are indicated, and these are discussed in the text. A third encounter at a distance of 2.1 m was recorded in the participant’s notes from 18:24 to 21:59 h; using an RSSI threshold of −60 dBm, this is a false positive.

A final example of participant data in encounter mode is shown in Fig. 12. This participant had four devices in the home and provided the recorded RSSI for one device (the primary device). The participant indicated two encounters and described other interactions between the four devices in their home. First, from 10:34 to 11:18 h, the primary device interacted with one other device and generated encounter ID f74b. The encounter was reported to be less than 2 m, and the RSSI was approximately −37 dBm, well above a threshold of −60 dBm; this is a true positive.

During the second encounter, from 12:25 to 12:58 h, the primary device was interacting with two other devices, recorded in encounter IDs 4b9c and e1ce. Then, an encounter with only one device, encounter ID e1ce, was continued from 13:01 to 14:44 h. Encounter ID 4b9c RSSI was greater than −60 dBm for the duration of the 12:25 to 12:58 h encounter, indicating another true positive. Additionally, the RSSI for encounter 4b9c was substantially different from the baseline state. However, using a threshold of −60 dBm, the RSSI from encounter ID e1ce recorded two false positives prior to the reported encounter.

Finally, encounter ID 3ba1 was an interaction with a device located at 2.1 m from the primary device from 18:24 to 21:59 h, and the measured RSSI was approximately −55 dBm. Using a threshold of −60 dBm to indicate distances less than 2 m, this is a false positive.

Based on this data set, the Bluetooth signal interaction between devices may not be sufficiently accurate in complex environments (*e.g.*, homes and office buildings) to assess significant encounters (*e.g.*, within the same airspace and within 2 m for a specific duration of time). When the devices were used in encounter mode, there was no control over the device orientation, and the relative positions of antennae can impact the received signal. Additionally, we observed that the baseline RSSI for a particular pair of devices can vary, such as the different RSSI values measured for encounter ID 4b9c and encounter ID f74b in Fig. 12C.

## 5 Discussion and Conclusion

Here, we have detailed the design, construction, and testing of a low-cost, highly privacy preserving wearable exposure notification device. The device operates independently of a smartphone, which enables encounter notification for persons without access to phones and could be more privacy preserving than a smartphone-based protocol (including a lack of location data). The method (devices and analyses) was not able to reliably use RSSI as a proxy for distance between pairs of devices; the accuracy of RSSI as a proxy for distance decreased dramatically outside of the idealized conditions. In particular, some orientations of the device resulted in low RSSI (< −60 dBm), even when the device was within 2 m of another device, possibly due to the uncontrolled orientation of the antennae when using the devices. While RSSI could not accurately predict a distance, it still may be useful for identifying a contact with some potential for transmission of an airborne virus, either with improvements to the device or for use in more defined situations.

Several improvements to the design and algorithm are necessary before widespread adoption could be encouraged. For example, channels 37, 38, and 39 did receive different signal levels depending on the orientation of the devices. In addition, since this design is not constrained to a smartphone, it is possible to use all 40 Bluetooth channels rather than just these three. Algorithmic development would be necessary to determine how to integrate information across channels [18–20]. With these improvements, the accuracy of RSSI to estimate distance could improve. Similarly, a different antenna design could improve the accuracy of the RSSI-based distance estimate. Finally, including an additional sensing method could improve distance estimation. For example, ultrasonic sensing (Appendix A) could be used to estimate distance and may be more accurate than RSSI at distances less than 2 m [21–22]. The inclusion of additional sensors, which could improve accuracy, would likely increase cost and decrease battery life.

Even an imperfect device, as presented here, could still be useful for some exposure notification methods, especially those that are not dependent on a strict 2 m definition. For example, aerosolized viruses can spread through the air farther than 2 m and may build up in enclosed spaces. Knowing that two individuals were near each other without being able to infer an accurate and precise distance may still be valuable for identifying a potential contact.

Beyond the COVID-19 pandemic, these devices could be used to understand disease spread and human or animal interaction. Data on how people interact in buildings and spaces (*i.e.*, encounter metrics) could be used to design buildings and spaces to be more resistant to disease spread. Additionally, agricultural communities can exhibit disease spread, and these low-power devices that are independent of smartphones could be used to understand animal interactions to limit disease spread.

While the device presented here is not ready for widespread use, the authors encourage others to continue to develop and test such devices, especially given the continued duration of the COVID-19 pandemic and the potential use of the devices in other settings.

## Figures and Tables

**Fig. A1:**
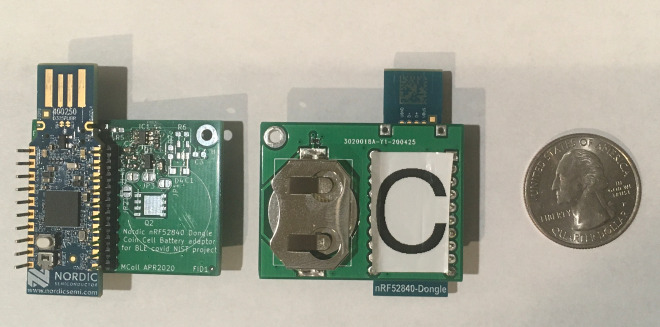
Photograph of development boards mounted to an accessory board. The assembly on the left shows the development board mounted (blue printed circuit board) to a custom accessory board (green printed circuit board). The assembly on the right is the underside of the accessory board that has a coin cell battery clip to power the assembly. A U.S. quarter dollar coin is shown for scale.

## 6 Appendix A

After initial tests with several development boards [23], and prior to using the Silicon Labs Thunderboard, we used the development board for the nRF52840 chip available from Nordic Semiconductor. The board can communicate using Bluetooth 5 [10] and is projected to operate between one week and one month using an inexpensive coin cell battery. It also has a suitably powerful central processing unit to calculate cryptographic hashes in a time fast enough for our exposure notification protocol (<100 ms). To create the wearable devices for the automatic exposure notification system, the development boards were paired with a custom accessory board with external memory for storing encounters and a coin cell battery holder. The development board and accessory board are shown in Fig. A1.

In addition to proximity detection using radio waves, it is possible to determine the distance between two devices using ultrasonic ranging. We started to explore the possibility of combining Bluetooth and ultrasonic ranging in one device, but we did not thoroughly test the ultrasonic ranging. We briefly describe our low-cost, low-power system here.

For the ultrasonic ranging, we added an inexpensive piezo buzzer to the Bluetooth development board using a small expansion connector on the board. We also used two pulse density modulation (PDM) microphones that were built into the Silicon Labs development board. The piezo buzzer and PDM microphones were able to transmit and record at ultrasonic frequencies between 25 kHz and 50 kHz. To obtain an estimate of the distance between two devices, one device would be the ultrasonic transmitter, and the other device would be the receiver. The transmitter would emit a Bluetooth signal and play a short ultrasonic pulse at a fixed frequency for 2 ms. The receiver would measure the time between receiving the Bluetooth signal and the slower audio signal. To reject background noise at the receiver, a high-pass filter was implemented in software to pass only sounds above 20 kHz. In addition, to further mitigate the effects from background ultrasonic noise from other device pairs that may be nearby, the transmitter would randomly pick a frequency between 25 kHz and 50 kHz for the short ultrasonic pulse. The receiver would only record signals at that frequency. As a result, many pairs of devices could be transmitting and receiving ultrasonic signals without interfering with each other. In the future, ultrasonic ranging capabilities could provide supplemental information to RSSI level, making the distance information more accurate.
